# HEVnet: a One Health, collaborative, interdisciplinary network and sequence data repository for enhanced hepatitis E virus molecular typing, characterisation and epidemiological investigations

**DOI:** 10.2807/1560-7917.ES.2019.24.10.1800407

**Published:** 2019-03-07

**Authors:** Annemieke Christine Mulder, Annelies Kroneman, Eelco Franz, Harry Vennema, Anna D. Tulen, Johanna Takkinen, Agnetha Hofhuis, Cornelia Adlhoch, Members of HEVnet

**Affiliations:** 1 Centre for Infectious Disease Control, National Institute for Public Health and the Environment (RIVM), Bilthoven, the Netherlands; 2 European Centre for Disease Prevention and Control (ECDC), Stockholm, Sweden; 3 These authors contributed equally to this article and share last authorship; 4 The members of HEVnet are listed at the end of the article

**Keywords:** hepatitis E virus, molecular typing, database, genotype, Europe, HEVnet, food-borne infections, zoonotic infections, viral infections, hepatitis E, HEV

## Abstract

Hepatitis E virus (HEV) is a common cause of acute hepatitis worldwide. In Europe, HEV is a zoonosis transmitted via contaminated pork meat or other pork food products. Genotype 3 is the most prevalent HEV type in the animal reservoir, as well as in humans. Despite an increased incidence of hepatitis E across Europe, much remains unknown about its spread, sources and transmission routes. A One Health approach is crucial to better understand the (molecular) epidemiology of HEV. HEVnet was established in April 2017 as a network and database for sharing sequences and accompanying metadata collected from human, animal, food and environmental sources. HEVnet members working in the public health, veterinary health, food, environmental and blood safety sectors have submitted 1,615 HEV sequences from nine countries as at January 2019. Most are from humans (89%), and sequences of animal (5%), food (6%) or environmental (0.3%) origin are rare. Metadata for human sequences capture mostly sex (93%), year of birth (92%) and sampling (100%); data on region of sampling (37%) and clinical information (hospitalisation 27%, symptoms 20% or mortality 8%) are limited. HEVnet aims to expand into a global network capable of performing cross-sectoral and supranational studies, with a joint repository of molecular and epidemiological data on HEV.

## Introduction

Hepatitis E virus (HEV) is one of the most common causative agents of acute hepatitis worldwide, with four major genotypes (1–4) affecting humans. Genotypes 1 and 2 are endemic in tropical and subtropical regions of Africa and Asia, where hepatitis E is a waterborne disease associated with sporadic cases and large outbreaks via faecal-oral transmission or contaminated water. HEV1 and HEV2 infections in Europe and the United States (US) are travel associated [1]. In Europe, hepatitis E is considered a zoonosis transmitted via contaminated pork meat or other pork food products. Genotype 3 and, to a lesser degree, genotype 4 are prevalent in the animal reservoir (pigs, wild boar and deer), as well as in humans [2]. There are occasional reports of HEV transmission through other routes, such as shellfish, salad or vegetables contaminated by sewage water carrying HEV from infected animals or humans [2-7]. Infection through contaminated blood products or other substances of human origin have been reported, and some European countries (the United Kingdom, followed by Ireland and the Netherlands) have implemented comprehensive testing of blood donations to reduce infections among patients who are vulnerable to developing chronic hepatitis E [8]. However, food appears to be the main transmission route in Europe.

A One Health approach is necessary to understand and mitigate HEV transmission via substances of human origin, animals, food and the environment [2]. Collaboration across disciplines is also crucial for the implementation of prevention and control measures that benefit public health, as HEV does not cause disease in animals and is therefore not considered a relevant veterinary issue requiring animal disease-control measures [9,10].

According to hepatitis E-specific surveillance systems in 20 European Union (EU)/European Economic Area (EEA) countries, more than 20,000 cases were reported between 2005–15, of which most were autochthonous [8,11]; more than 50% of the cases with known clinical information were reported from hospital settings and five countries reported a total of 28 fatal cases related to a HEV infection during this period [8]. Based on these reports, the European Centre for Disease Prevention and Control (ECDC) identified a need to support activities related to the investigation and assessment of HEV in the EU/EEA.

During the first ECDC HEV expert meeting in December 2015—attended by nominated scientists working on HEV-related topics in the EU/EEA, the World Health Organization (WHO) and the European Food Safety Agency (EFSA)—the development of a joint database of HEV sequences with metadata to support molecular epidemiological investigations was set as a priority, as molecular sequences provide more discriminatory power than only genotypes and subtypes of the virus [8,12]. The subsequent year, the ECDC and its HEV expert group further developed the concept of the database in collaboration with The National Institute for Public Health and the Environment (RIVM). RIVM already hosted pathogen-specific online databases such as HAVnet (for hepatitis A viruses) and NoroNet (for noroviruses), which are tools that have supported international investigations of outbreaks and molecular trends through standardisation and sharing of protocols [13-15].

This manuscript aims to introduce HEVnet (http://www.hevnet.nl), an international, cross-disciplinary database of HEV sequence data retrieved from different sources (humans, animals, the environment and food) and accompanied by relevant metadata for analysing the molecular epidemiological relationships between circulating HEV strains.

## The HEVnet database 

The HEVnet database is a password-protected online environment for sharing and analysing HEV sequence data accompanied by metadata. The metadata provide information on the specimen origin (country of sampling, date, type), sample origin (human, food, environmental or animal), patient (sex, year of birth, symptoms, hospitalisation, underlying conditions, etc.), behavioural exposure (food worker, animal contact, wastewater contact, other) and clinical parameters such as organ transplant or blood transfusion. All data are anonymised to ensure that the original source of the sample (e.g. an individual) cannot be traced, as this type of data is subject to the European General Data Protection Regulation [16]. Upon uploading, all sequences are automatically typed by a publicly available, curated, phylogenetic typing tool (https://www.rivm.nl/mpf/typingtool/hev) in order to secure standardised genotype and subtype assignment using the full-length sequences of reference viruses for HEV typing and subtyping proposed by Smith et al. in 2016 [17,18], supplemented with representatives of tentative new subtypes.

Data can be queried, analysed and visualised as pie charts, geographical maps and phylogenetic trees, using a set of analysis modules within the protected online working space. Members can retrieve whole sequences or a queried selection of the sequences in FastA format for phylogenetic analyses. The metadata, with a link to the sequences, can be downloaded in Excel format for data analyses. The combination of phylogenetic and epidemiological data enables virological, epidemiological or combined investigations.

## HEVnet network

The HEVnet network consists of HEV experts, such as virologists or epidemiologists in the public health, veterinary health, food, environmental and blood safety sectors. Participation in HEVnet is voluntary. A data confidentiality agreement grants membership and use of the database. Members are expected to respect a ‘quid pro quo’ principle in using the HEVnet data. The complete dataset is available to HEVnet members, and data ownership remains with the data provider. Compliant with the GDPR, and to promote research benefiting public health, all sequence data with a restricted set of associated metadata become publicly available at a time specified by the data provider, but no later than 18 months after submission.

## HEVnet objectives

At the first HEVnet meeting in October 2017, 16 network members, RIVM’s HEVnet team and representatives of ECDC and EFSA agreed that the objectives of HEVnet are to use molecular typing to assess trends in circulating HEV genotypes and subtypes in humans at an international level and to analyse the distribution of HEV subtypes in humans for better understanding of the epidemiology (e.g. characteristics) of the affected populations and the geographical relationships between viruses. Furthermore, HEVnet supports the investigation of clusters across countries. The pooled analysis of sequences across disciplines generates evidence about the relationships between isolates from human, animal, food and environmental specimens, and may help to identify sources of infection and transmission routes. Increased availability of sequence information should enable more in-depth virological analysis to study viral evolution. Linking of case-based patient data with molecular information could also support clinical studies on the pathogenicity and severity of viral types and subtypes, as well as the association of genotypes and clinical manifestations.

Table 1 outlines the objectives of HEVnet, as well as the data requirements and expertise needed to meet these objectives, such as standardisation of the generating and reporting of sequence data. As a first step, the HEVnet members agreed that a minimum sequence length of 300 nt within ORF2 is required for robust analyses of HEV. Further, results relevant to risk assessments or clinicians will be communicated to the respective stakeholders, e.g. EFSA and ECDC.

**Table 1:** Overview of data and experts required to reach the objectives of HEVnet

Requirements		1. Distribution and trends of circulating human HEV subtypes^a^	2. Molecular epidemiology^b^	3. Cluster investigation^c^	4. Relationship of viruses for source attribution^d^	5. Virus evolution and spread^e^	6. Clinical virology: severity of viral (sub)types^f^
Data	Human origin sequences and typing results according to standardised nomenclature, with information on time and place	Y	Y	Y	Y	Y	Y
	Animal/food/environmental origin sequences and typing results according to standardised nomenclature, with time and place	N	N	N	Y	Y	N
	Sequences of an agreed genomic region: minimum length of 300 nt within ORF2	N	Y	Y	Y	Y	Y
	Standardised metadata: demographic and epidemiological	N	Y	Y	Y	N	N
	Standardised metadata: clinical data	N	N	N	N	N	Y
	Timely reporting	N	N	Y	Y	N	N
Expertise	Public health epidemiologists or bioinformaticians	Y	Y	Y	Y	Y	Y
	Clinical virologists	N	Y	Y	Y	Y	Y
	Food/veterinary/environmental virologists	N	N	N	Y	Y	N
	Physicians	N	N	Y	N	N	Y

HEV: hepatitis E virus.

^a^ Objective 1: to use molecular typing to assess the distribution and trends of HEV genotypes and subtypes circulating in humans.

^b^ Objective 2: to analyse the distribution of subtypes in humans, leading to a better understanding of the underlying epidemiology of HEV.

^c^ Objective 3: to combine human sequences with provided metadata of human cases, for cluster investigation.

^d^ Objective 4: to trace the most likely reservoirs and sources of HEV by identifying connections between human and non-human (food, animal and environmental) samples via source attribution studies.

^e^ Objective 5: to do population genetics studies into the spread and evolution of HEV.

^f^ Objective 6: to assess the pathogenicity of strains through assessment of relationships between viral subtypes and severity of disease, looking at symptoms, hospitalisation and mortality.

## Current and future uses of the HEVnet database

Since the system was launched in April 2017, 41 individual accounts have been created in HEVnet for experts from 15 countries: Austria, Belgium, Denmark, England and Wales, Estonia, France, Germany, Ireland, Italy, the Netherlands, Portugal, Spain, Sweden, Switzerland and the US. With the exception of the one member from the US, all current experts in the network are from Europe. However, HEVnet aims to expand this collaboration into a global network. 

HEVnet has the potential to become an important resource for the scientific community working in the field of HEV. With the currently available sequences, patterns in circulating HEV subtypes can be analysed to identify and propose new reference viruses and to harmonise the classification and nomenclature of genotypes and subtypes across countries. Future work will include the standardisation of sequencing protocols, an update of the reference set with tentative new subtypes (focussed on genotype 3) and analyses of the submitted data.

Table 2 provides an overview of the metadata reported for 1,615 sequences submitted by 15 institutes in nine countries for the period April 2017–January 2019. These sequences mainly originate from human cases (89%), and are rarely of animal (5%), food (6%) or environmental origin (0.3%). The number of sequences obtained from animals, food and the environment is not yet sufficient to investigate the HEV relationships across these disciplines, which is crucial for understanding the epidemiology of HEV. HEVnet encourages HEV experts from the veterinary, food and environmental sectors to join the network and support its growth by disseminating this invitation and connecting with projects of One Health initiatives, such as the One Health European Joint Programmes (https://onehealthejp.eu/) and the Med-Vet-Net Association (http://mvnassociation.org/medvetnet/index.php).

**Table 2:** Overview of metadata reported for 1,615 sequences submitted to HEVnet, April 2017–January 2019

Metadata with sequences	N	% (of 1,615)
Sample origin is reported	1,615	100%
Human origin	1,443	89%
Animal origin	75	5%
Food origin	92	6%
Environmental origin	5	0%
Year of sampling is reported	1,615	100%
Sequencing method is reported	1,592	99%
Region of sampling is reported (province or higher resolution)	562	35%
**Metadata with sequences of human origin**	**1,443**	**%** **(of 1,443)**
Sequencing method is reported	1,420	98%
Patient sex is reported	1,349	93%
Patient year of birth is reported	1,332	92%
Year of sampling is reported	1,443	100%
Hospitalisation ‘yes/no’ is reported	391	27%
Symptoms are reported	293	20%
Mortality ‘yes/no’ reported	109	8%
Region of sampling is reported (province or higher resolution)	528	37%

Molecular epidemiological analyses focussing on human clinical data, including sequences from blood donors and patients with chronic HEV infection, might help to identify and better understand relationships between viruses, with regard to severity and associated risk factors. HEVnet allows collecting an array of clinically relevant parameters, such as duration and nature of symptoms, hospitalisation, comorbidity and treatment outcomes. However, as shown in Table 2, the majority of submitted sequences of human origin contain only metadata on patient sex (93%), year of birth (92%) and year of sampling (100%). Far fewer sequences have metadata on the region of sampling (37%) or clinical information such as hospitalisation (27%), symptoms (20%) or mortality (8%). Improving the completeness of metadata in HEVnet could be accomplished if future studies link more variables of metadata to submitted sequences. For example, the Netherlands included a broad range of anonymised metadata when they submitted all available HEV sequences from a recent nationwide patient control study into risk factors of HEV [19]. Authors of future studies in which HEV sequences are collected together with metadata are encouraged to connect with HEVnet so they can benefit from the network collaboration by comparing their newly collected sequences to the worldwide repository. The availability of large datasets and full-length sequences will enable the refinement of current subgenotype classification and the identification of new divergent subtypes in both humans and animals, over time.

## Conclusion

Using a joint repository of molecular and epidemiological data, HEVnet enables cross-sectoral and supranational collaborations to conduct molecular epidemiological investigations on HEV. A One Health approach is necessary to understand and mitigate HEV transmission via food, animals, substances of human origin and the environment. To strengthen the HEVnet database as an international One Health platform, it is necessary that more experts in the veterinary, food and environmental sectors join the network and submit their sequences with metadata. HEV experts across the world are invited to join the network by sending an email request (hevnet@rivm.nl) with a short explanation of what they would like to contribute to the network and the establishment of a worldwide reference set of HEV sequences.

## Members of HEVnet

Stephan Aberle, Medical University of Vienna, Vienna, Austria

Lorenzo Subissi, Sciensano, Brussels, Belgium

Vanessa Suin, Sciensano, Brussels, Belgium

Sofie Midgley, Virus surveillance and research, Statens Serum Institut, Copenhagen, Denmark

Tatiana Kuznetsova, Department of Virology and Immunology, National Institute for Health Development, Tallinn, Estonia

Jacques Izopet, Federative Intitute of Biology, National Reference Center for Hepatitis E virus, Toulouse, France

Nicole Pavio, French Agency for Food, Environmental and Occupational Health and Safety (ANSES), Maisons-Alfort, France

Christine Baechlein, University of Veterinary Medicine Hannover, Hannover, Germany

Sally A. Baylis^a^, Paul-Ehrlich-Institut (PEI), Langen, Germany

Victor M. Corman, Institute of Virology Charité – Universitätsmedizin Berlin, Berlin, Germany

Mirko Faber^a^, Robert Koch-Institut, Berlin, Germany

Reimar Johne, German Federal Institute for Risk Assessment (BfR), Berlin, Germany

Christel Kamp, Paul-Ehrlich-Institut (PEI), Langen, Germany

Juergen J. Wenzel, Institute of Clinical Microbiology and Hygiene, Regensburg University Medical Center, Regensburg, Germany

Suzie Coughlan, National virus reference laboratory, University College Dublin, Dublin, Ireland

Ilaria Di Bartolo, Istituto Superiore di Sanità, Rome, Italy

Roberto Bruni, Istituto Superiore Di Sanità, Rome, Italy

Anna Rita Ciccaglione^a^, Istituto Superiore di Sanità, Rome, Italy

Anna Rosa Garbuglia, Laboratory of virology INMI Spallanzani IRCCs Italy

Elisabetta Suffredini, Department of Food safety, Nutrition and Veterinary public health, Istituto Superiore di Sanità, Rome, Italy

Ingeborg Boxman, the Netherlands Food and Consumer Product Safety Authority (NVWA), the Netherlands

Boris Hogema, Sanquin Blood Supply Foundation, Amsterdam, the Netherlands

Wim van der Poel, Wageningen BioVeterinary Research, Wageningen, the Netherlands

Hans Zaaijer^a^, Sanquin Blood Supply Foundation, Amsterdam, the Netherlands

Rita de Sousa^a^, Instituto Nacional de Saúde Doutor Ricardo Jorge, Lisbon, Portugal

Branko Velebit, Institute of Meat Hygiene and Technology, Serbia

Ana Avellón^a^, Viral Hepatitis Reference and Research Laboratory National Center of Microbiology Carlos III Health Institute, Madrid, Spain

Maria Buti, Hospital Universitario Valle Hebron, Barcelona, Spain

Rosina Girones, Section of Microbiology, Virology and Biotechnology Department of Genetics, Microbiology and Statistics Faculty of Biology University of Barcelona, Barcelona, Spain

Josep Quer, Vall d'Hebron Institut Research, Barcelona, Spain

Frederik Widén, National Veterinary Institute (SVA), Uppsala, Sweden

Heléne Norder, Sahlgrenska University Hospital, Gothenburg, Sweden

Kristina Nyström, Sahlgrenska University Hospital, Gothenburg, Sweden

Claudia Bachofen, Institute of Virology, Vetsuisse Faculty of the University of Zürich, Zürich, Switzerland

Roland Sahli, Institute of microbiology, Centre Hospitalier Universitaire Vaudois and University of Lausanne, Lausanne, SwitzerlandHarry Dalton^a^, Royal Cornwall Hospital, Treliske, UK

Samreen Ijaz^a^, Public Health England, London, UK

Samantha Treagus, CEFAS, University of Exeter, UK

Michael Kulka, Center for Food Safety and Applied Nutrition, U.S. Food and Drug Administration, Unites States of America

Valentina Rizzi, European Food Safety Authority (EFSA), Unit of Biological Hazards and Contaminants, Parma, Italy

^a ^Also a member of the European Centre for Disease Prevention and Control (ECDC) HEV expert group.
